# Perioperative Considerations in a Patient With Oculopharyngeal Muscular Dystrophy: A Case Report

**DOI:** 10.7759/cureus.90496

**Published:** 2025-08-19

**Authors:** Pratima Bajaj, David Overholt, Mamta Chura

**Affiliations:** 1 Anesthesiology, Augusta University Medical College of Georgia, Augusta, USA

**Keywords:** anesthetic management, high aspiration risk, neuromuscular blockade sensitivity, oculopharyngeal muscular dystrophy, perioperative considerations

## Abstract

Oculopharyngeal muscular dystrophy (OPMD) is a rare, late-onset neuromuscular disorder that is classically characterized by dysphagia, ptosis, and proximal limb weakness. Although OPMD is typically mild in progression, it presents unique challenges in the perioperative setting due to pharyngeal muscle weakness, increased risk of aspiration, and sensitivity to neuromuscular blocking agents. Here, we discuss the anesthetic management of a 75-year-old male with OPMD undergoing an elective reverse total shoulder arthroplasty. Though preoperative pulmonary function testing showed preserved respiratory mechanics, the patient had previously reported dysphagia, voice changes, and fatigue. Due to the increased risk for perioperative complications, an anesthetic plan was developed with the primary goals of reducing aspiration risk, minimizing neuromuscular blockade, and facilitating a smooth emergence. For induction of anesthesia, total intravenous agents were used. Inhaled anesthetics were avoided to decrease the risk of residual respiratory depression. Endotracheal intubation was performed under video laryngoscopy after administering a reduced dose of rocuronium. Intraoperatively, train-of-four stimulation was utilized for close monitoring of neuromuscular blockade. Following partial recovery of consciousness after case completion, rocuronium was reversed with sugammadex. The patient was then extubated and monitored in the post-anesthesia care unit. No respiratory sequelae were noted. This case highlights the need for individualized anesthetic planning in patients with OPMD, especially in the context of airway protection and neuromuscular management. Avoiding succinylcholine, limiting non-depolarizing agents, and utilizing neuromuscular monitoring may help mitigate the risk of prolonged paralysis and respiratory compromise. As more aging patients with neuromuscular disorders present for surgery, it is essential to promote increased awareness of the specific anesthetic implications of OPMD. This case contributes to the limited body of literature guiding safe perioperative care in this patient population.

## Introduction

Oculopharyngeal muscular dystrophy (OPMD) is an autosomal dominant neuromuscular condition characterized by onset during adulthood and slowly progressive muscular weakness. OPMD is a rare condition, as the overall incidence of OPMD in the United States is approximately 0.001%, with a higher prevalence noted in populations of French-Canadian, Ashkenazi Jewish, and Spanish American descent [1]. Clinical manifestations of OPMD classically include bilateral ptosis, dysphagia, and proximal limb muscle weakness [1-3]. The pathophysiology of OPMD involves the abnormal expansion of GCN trinucleotide repeats within the polyadenylate-binding protein nuclear 1 (PABPN1) gene, resulting in the accumulation of misfolded PABPN1 proteins and the formation of intranuclear inclusions within muscle cells. These inclusions disrupt normal cellular functions and ultimately lead to progressive degeneration of the affected muscle fibers [4,5]. While the prognosis of OPMD is generally favorable for individuals with heterozygous GCN repeat expansions, as OPMD does not typically reduce life span, the quality of life for individuals with OPMD is significantly impacted in later years due to progressive dysphagia and muscle weakness [1].

Anesthetic management of OPMD patients necessitates careful perioperative consideration due to distinct clinical challenges, specifically the risk of aspiration and respiratory insufficiency due to oropharyngeal muscle weakness. Management thus requires meticulous airway management, proactive aspiration prophylaxis, and diligent postoperative respiratory monitoring [6,7]. Further, patients with OPMD also exhibit heightened sensitivity to neuromuscular blocking agents, so there is an associated risk of prolonged neuromuscular blockade. Continuous intraoperative neuromuscular monitoring is thus important to prevent delayed anesthetic emergence and the associated respiratory complications [8-10].

Given the paucity of literature that addresses anesthetic management protocols tailored to OPMD patients undergoing orthopedic procedures, it is necessary to individualize anesthetic plans based on the patient’s history and presentation. Systematic documentation and reporting of anesthetic practices in patients with OPMD will help facilitate the development of evidence-based guidelines to improve patient outcomes.

## Case presentation

A 75-year-old male, 80 kg, with OPMD was scheduled to undergo an elective reverse left shoulder arthroplasty due to chronic shoulder pain. He had previously failed extensive interventions, including a revision rotator cuff repair performed 10 years prior, physical therapy, subacromial joint injections, and stem cell treatments. Other past medical history included atrial fibrillation on apixaban, sick sinus syndrome with dual chamber pacemaker placement in September 2019, left cerebellar ischemic stroke in March 2022, obstructive sleep apnea, and borderline diabetes.

The patient’s OPMD diagnosis was recently genetically proven with an identified pathogenic PABPN1 mutation. The patient had an extensive history of chronic complaints of bilateral upper and lower extremity weakness, ptosis, choking with meals, and significant difficulty swallowing. He also noted a strong family history of OPMD in his father, paternal aunt, and both sisters; his children were not reported to be affected. Physical exam findings exhibited bilateral cheek weakness, tongue weakness, moderate neck flexor weakness, mild extensor weakness, mild lateral neck rotation weakness in both directions, and mild proximal upper extremity weakness bilaterally. Barium swallow testing showed moderate OPMD characterized by decreased hyolaryngeal elevation and anterior excursion that resulted in incomplete epiglottic inversion and vallecular residue with all bolus trials. Additionally, EMG testing showed possible myopathic units with spontaneous activity in the right deltoid. However, pathology results from a right deltoid biopsy two years ago indicated only rare acutely denervated atrophic fibers without significant myopathic changes.

Labs and imaging obtained on the day of the procedure were unremarkable. The patient received a single-shot preoperative left interscalene block at the surgeon’s request for management of postoperative pain. Routine monitors were attached in the operating room. After preoxygenation, he was induced with lidocaine 50 mg, propofol 200 mg, rocuronium 60 mg, and fentanyl 100 mcg. He was then successfully intubated on the first attempt with a McGrath size 3 blade and a size 7.0 endotracheal tube with a stylet. Appropriate placement was verified by auscultation, chest rise, and end-tidal CO₂ monitoring. The tube was secured at 23 cm at the lips on the right side. During the procedure, anesthesia was maintained with propofol and remifentanil infusions. Inhalational agents were avoided due to the risk of exaggerated hyperkalemia. The patient’s blood pressure was intermittently managed with a phenylephrine infusion. No invasive monitoring was required. At the end of the case, trial extubation was successful, and his postoperative course was uncomplicated. He was further monitored in the post-anesthesia care unit and admitted for an overnight stay for monitoring due to his comorbidities.

## Discussion

Anesthetic management of patients with OPMD is complex due to the disease’s hallmark symptoms of oropharyngeal and respiratory muscle weakness. Due to the significantly increased risk of aspiration, airway obstruction, and respiratory complications, heightened vigilance is required throughout the perioperative course [2].

Initially, preoperative assessment should include an evaluation of the degree of dysphagia, history of aspiration, and baseline respiratory function. Preparation for advanced airway interventions is also important. Intraoperatively, meticulous airway management is essential, and rapid sequence intubation should be considered if it is indicated by the patient’s history and symptoms [7]. Perhaps the most critical perioperative concern is aspiration risk since dysphagia and impaired airway protection are common in OPMD and can lead to aspiration pneumonia. Previously, the importance of preoperative assessment of dysphagia and perioperative aspiration has been emphasized [11,12].

In addition, local anesthesia may be preferable to general anesthesia for minor procedures, as it is associated with fewer respiratory complications and shorter recovery times compared with general anesthesia. The type of procedure may also help guide anesthetic choice since procedures that involve the upper airway carry a higher risk. A retrospective study of 114 surgeries in 92 OPMD patients found that respiratory complications, including aspiration, airway obstruction, and severe coughing, were more frequent after cricopharyngeal myotomy than after levator palpebrae resection. Local anesthesia was also associated with fewer complications and shorter recovery times than general anesthesia [6].

Patients with OPMD are also at risk for prolonged neuromuscular blockade and respiratory failure when they are exposed to neuromuscular blocking agents. In neuromuscular disorders, since pharmacokinetics and pharmacodynamics are altered, administration of neuromuscular blocking agents can result in an exaggerated response to or prolonged effects of these agents. This can lead to delayed recovery of muscle strength and an increased risk of postoperative respiratory complications such as hypoventilation and the need for reintubation [7,10]. Of note, depolarizing agents such as succinylcholine should also be avoided in OPMD because of the increased risk of life-threatening hyperkalemia, rhabdomyolysis, and malignant hyperthermia. The American Society of Anesthesiologists specifically recommends against the use of succinylcholine in patients with neuromuscular disorders due to these risks. Fatal hyperkalemic cardiac arrest and malignant hyperthermia have previously been documented in the literature in patients with underlying myopathies who were administered succinylcholine [10,13].

As such, nondepolarizing neuromuscular blocking agents are preferred, but these agents should also be used with caution. Quantitative neuromuscular monitoring should be utilized to effectively titrate dosing and detect residual blockade. Previously, prolonged effects of and increased sensitivity to these agents have been reported. Common complications include unexpected ICU admissions and delayed extubation [10,14]. For the reversal of nondepolarizing agents in neuromuscular disease, sugammadex is recommended since it provides rapid reversal while reducing the risk of residual paralysis and respiratory failure. Recent case series and reviews have repeatedly demonstrated the safety and efficacy of sugammadex in this population by highlighting a lower incidence of postoperative complications compared to traditional reversal agents [10,14,15].

In the postoperative period, intensive and prolonged monitoring is critical, with a focus on early detection and management of respiratory complications, aspiration, and residual neuromuscular blockade. Continuous pulse oximetry and capnography are also recommended. A close eye should be kept on subtle signs of aspiration, such as cough and increased secretions, since aspiration can quickly progress to pneumonia or respiratory failure. Airway clearance techniques, including manual or mechanical cough assistance, should also be considered for patients with ineffective cough, as recommended by the American College of Chest Physicians. In addition, opioids and sedatives should be used carefully to avoid further respiratory depression. Further, early mobilization, chest physiotherapy, and close multidisciplinary collaboration should be promoted to optimize outcomes [8,10,15].

In this case, individualized anesthetic care for our patient with OPMD was the key consideration. Given the risks associated with pharyngeal muscle weakness, impaired airway protection, and sensitivity to neuromuscular blocking agents, the anesthesia team employed careful planning to ensure a smooth perioperative course. The use of total intravenous anesthesia, avoidance of depolarizing agents, and close neuromuscular monitoring allowed for safe induction and emergence, while reversal with sugammadex helped reduce the chance of residual blockade. The patient’s uncomplicated outcomes reflect the value of anticipating and planning for the potential perioperative complications associated with OPMD.

The literature on anesthetic management in OPMD is sparse and comprises mainly small retrospective studies and isolated case reports without any large prospective trials or disease-specific guidelines. This paucity of data necessitates individualized, multidisciplinary perioperative planning and a high index of suspicion for complications.

## Conclusions

Due to its characteristic involvement of pharyngeal and respiratory muscle involvement, OPMD poses distinct anesthetic challenges. Among the many other potential issues that may arise perioperatively, the risk of aspiration and increased sensitivity to neuromuscular blocking agents particularly necessitate a cautious and individualized approach, even in patients who have normal functioning at baseline. With the limited availability of formal guidelines for anesthetic management of such conditions, the accumulation of real-world clinical experiences remains crucial in enhancing our understanding of perioperative risks and management.
